# Spatio-temporal responses of predators to hyperabundant geese affect risk of predation for sympatric-nesting species

**DOI:** 10.1371/journal.pone.0221727

**Published:** 2019-08-28

**Authors:** Scott A. Flemming, Erica Nol, Lisa V. Kennedy, Audrey Bédard, Marie-Andrée Giroux, Paul A. Smith

**Affiliations:** 1 Environmental and Life Sciences, Trent University, Peterborough, Ontario, Canada; 2 Biology Department, Trent University, Peterborough, Ontario, Canada; 3 Departement de Chimie et de Biochimie, Universite de Moncton, Moncton, New Brunswick, Canada; 4 National Wildlife Research Centre, Environment and Climate Change Canada, Ottawa, Ontario, Canada; Universidad Miguel Hernandez de Elche, SPAIN

## Abstract

The Arctic is undergoing rapid changes, with anthropogenic shifts in climate having important and well-documented impacts on habitat. Populations of predators and their prey are affected by changing climate and other anthropogenic factors, and these changing trophic interactions could have profound effects on breeding populations of Arctic birds. Variable abundance of lemmings (a primary prey of generalist Arctic predators) and increasing abundance of light geese (Lesser Snow and Ross’ Geese; a secondary prey) could have negative consequences for numerous sympatric shorebirds (an incidental prey). Using 16 years of predator-prey observations and 13-years of shorebird nest survival data at a site near a goose colony we identify relationships among geese, lemmings, and their shared predators and then relate predator indices to shorebird risk of nest predation. During two years, we also placed time-lapse cameras and artificial shorebird nests at increasing distances from a goose colony to document spatial trends in predators and their effect on risk of predation. In the long-term data, yearly indices of light geese positively influenced indices of gulls and jaegers, and shorebird nest predation rate was negatively correlated with jaeger and fox indices. All three predator indices were highest near the goose colony and artificial nest predation probability was negatively correlated with distance from goose colony, but these effects were less apparent during the second year. Combined, these results highlight the variation in predator-mediated interactions between geese and shorebirds and outline one mechanism by which hyperabundant geese may be contributing to local or regional declines in Arctic-nesting shorebird populations.

## Introduction

The Arctic is undergoing rapid changes with anthropogenic-driven shifts in climate having important and well-documented impacts on habitat quality, quantity, and spatial distribution (e.g., [1]). These changes could significantly influence populations of Arctic-breeding birds, the most diverse and vertebrate taxa of the circumpolar Arctic [2]. Northward shifts in vegetation communities have already influenced the availability of nesting habitat for sub-arctic breeding shorebirds [3], and advances in peak prey availability have created a mismatch with the phenology of chick hatch [4], and lower growth and chick survival in some [5,6] but not all cases [4,7,8]. While at large scales these changes have the potential to alter bird distribution, phenology, and demography, at smaller spatial scales the trophic interactions among predators and their prey may play a more dominant role in structuring communities [9,10]. These predator-prey interactions can have cascading effects on the populations of sympatric birds.

In northern regions, arctic foxes (*Vulpes lagopus*) and jaegers (Parasitic, *Stercorarius parasiticus* and to a lesser extent, Long-tailed Jaeger, *S*. *longicaudus*) depredate shorebird nests “incidentally” [11,12] lemmings (*Dicrostonyx spp*., *Lemmus spp*.) and the eggs and chicks of larger birds such as geese are the primary or secondary prey, and shorebird nests may be depredated opportunistically when they are encountered [13]. Nonetheless, these predators constitute the primary cause of nest failure for shorebirds across the Arctic [12,14,15]. Thus, lemmings, goose eggs, and goslings interact with shorebirds indirectly by influencing the abundance and behaviour of their shared predators.

Predator populations may exhibit numerical responses (increases in abundance) to lemming peaks [16,17] and more specifically, aggregative responses [17] to goose colonies, in both cases resulting in higher incidental predation of nearby shorebird nests. Alternatively, predators may switch their search efforts between prey items (functional response; [18], or become satiated during lemming peaks or within goose colonies, alleviating predation pressure on shorebird nests [19,20]. Local or regional changes in the availability of lemmings and/or geese could therefore dramatically affect the risk of predation and reproductive success of tundra-nesting shorebirds.

In the last 30 years climate-induced changes in environmental conditions have resulted in decreases in the frequency and amplitude of lemming peaks at some northern sites ([21–23] but see Ehrich et al. *in press*). Five-fold decreases in peak magnitude have been reported in Greenland [21]. By contrast, North American populations of Greater (*Chen caerulescens atlantica*) and Lesser Snow (*C*. *c*. *caerulescens*), and Ross’ Geese (*Chen rossii*; hereafter collectively referred to as ‘light geese’) have increased exponentially in abundance over the last 60 years [24,25], in response to food subsidies on their wintering grounds [26]. Band-recoveries now suggest that the adult midcontinent lesser snow goose population may be greater than 15 million individuals, an estimate significantly higher than the ~2.5 million estimated in the early 1970s [27].

Decadal declines and increases in the abundances of lemmings and light geese, respectively, could have negative consequences for numerous Arctic-breeding shorebirds whose populations have declined over this same period [28–30]. Although these declines could arise from a multitude of factors across shorebirds’ large ranges, reproductive success for Arctic-breeding shorebirds is low at some locations (e.g., [31,32]) and theoretical and empirical studies have demonstrated that breeding failure has the potential to explain shorebirds’ negative population trends [9,33].

Despite low biodiversity in comparison to lower latitudes, Arctic systems are governed by complex trophic interactions among shorebirds, lemmings, and geese. Interactions mediated through shared predators may vary regionally depending on guilds of predators and prey, or climate. The objectives of our study were therefore to identify the numerical and functional responses of generalist predators to varying prey availability (i.e. lemming availability and goose colony presence), and relate these responses to predation risk for shorebird nests in the eastern Canadian Arctic. Using long-term field observations from a study site near a light goose colony, we identify temporal trends in predator-prey indices over a 16-year period and relate these trends to survival of shorebird nests. We then used 24-hour time-lapse cameras in concert with artificial shorebird nests both placed in plots situated at increasing distances from the goose colony to record spatial variation in predator indices and experimentally test for variation in predation risk in relation to the presence of nesting light geese.

## Materials and methods

### Study sites and plots

**Fig 1:**
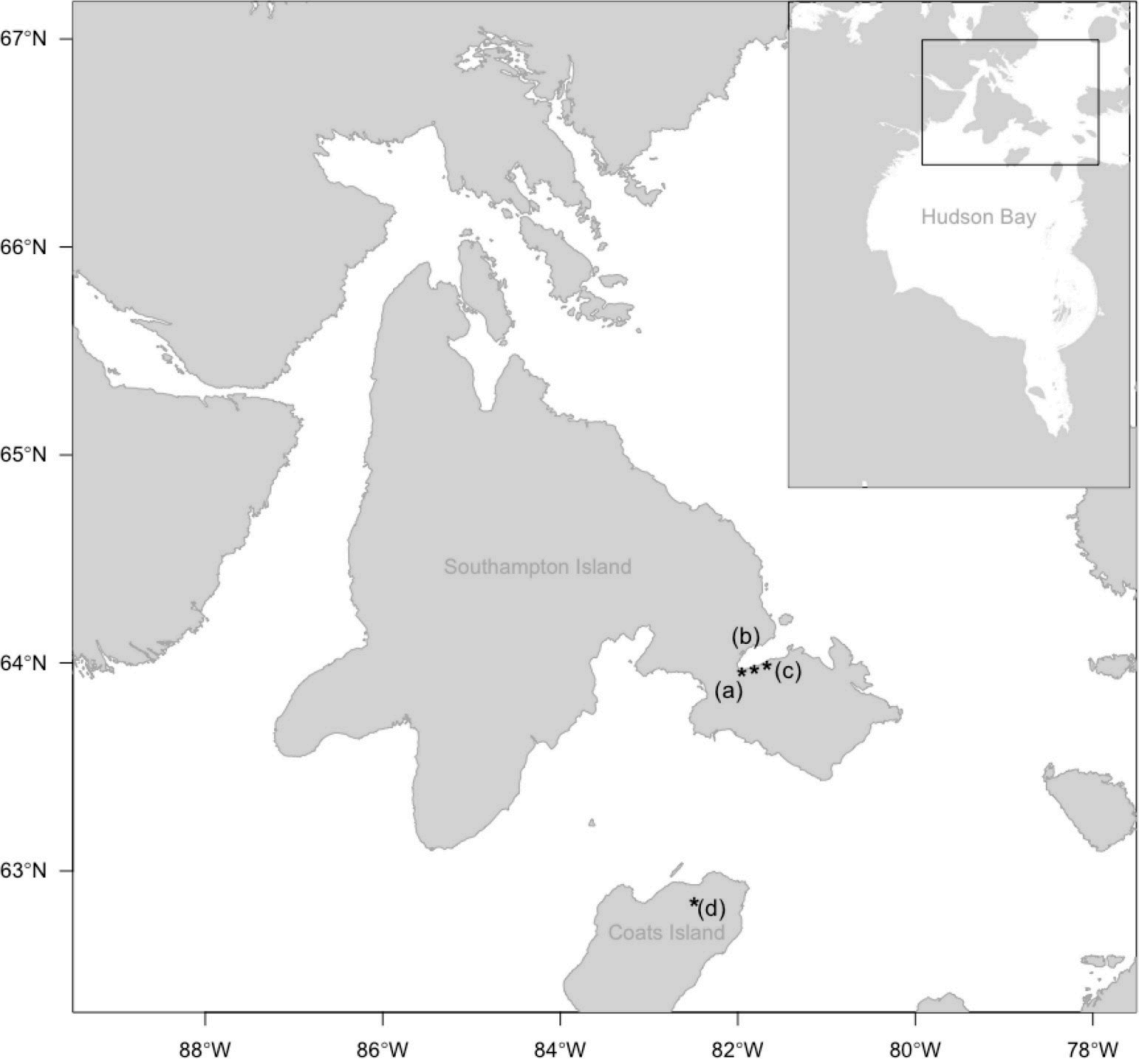
Map of study region. Study plots (a) Within, (b) Near, and (c) Far (East Bay Mainland) from a goose colony, and (d) on Coats Island, Nunavut.

We conducted research within three primary study sites and four plots in the eastern Canadian Arctic. The first study site is situated within a Lesser Snow Goose colony in the East Bay Migratory Bird Sanctuary (EBMBS) on Southampton Island, Nunavut (Fig 1). The populations of geese within this colony and an adjacent colony to the southwest (the “Coral Harbour” colony) have increased from 156,700 breeding birds in 1997 to 289,700 in 2014 ([25], J. Leafloor, *unpublished*). The second site, the East Bay Mainland Shorebird Camp (hereafter East Bay Mainland), is a long-term shorebird study site initiated in 1999 and is also situated within the EBMBS ~10km from the goose colony. Although the site is not typically used for breeding by light geese, family groups use the site extensively later in the season for foraging. The third study site, the Coats Island Shorebird Camp, is situated on Coats Island approximately 135km south of Southampton Island. Light geese do not breed regularly at this study site and lemmings are absent from the island [34].

To gauge spatial effects of light goose presence on predator indices and risk of predation we established four 10-12ha plots at increasing distances from the goose colony by drawing coordinates in a random-stratified fashion. The plots on Southampton Island were situated 0km (Within: 2 plots), 3-4km (Near: 2 plots) and 8-10km (Far: 3 plots) from the goose colony and predator indices and predation risk were compared to those from plots established on Coats Island (3 plots). We conducted fieldwork at these three sites during the shorebird breeding season from early-June to late-July, in 2015–2016. We also recorded a subset of variables (see below) at the East Bay Mainland site in 2000–2016.

### Study design

Our main objectives were to determine whether indices of predator abundance and activity responded to temporal and spatial variation in light goose and lemming abundance, and whether these changes affected the predation rates of shorebird nests. To identify temporal changes in the abundance and activity of predators and their prey, we generated group-specific indices (see below) from daily observations (hereafter observational predator-prey indices) at East Bay Mainland from 2000–2016 (We did not collect long-term observational indices at our other study sites). We then used shorebird nest survival at East Bay Mainland from 2004–2016 to relate predator-prey abundance and activity indices to risk of predation.

To identify spatial trends in predator abundance and activity, in 2015 and 2016 we generated predator indices using time-lapse cameras (hereafter camera predator indices) placed within the four study plots established at increasing distances from the goose colony. Measuring the spatial patterns in shorebird nest predation was challenging, in that too few shorebird nests were found Within and Near the goose colony for statistical analyses; presumably because of reduced nesting densities and nest survival in these heavily goose-affected areas. Therefore, in 2015 and 2016, we placed artificial nests within each of the four study plots to relate predation risk to spatial variation in camera predator indices. The Animal Care Committee of Environment and Climate Change Canada approved our procedures, and permits were granted from Federal and Territorial Governments (e.g., NUN-SCI-14-05, WL2016-053). No animals were handled during this study and there were no ethical concerns regarding animal welfare. All research was conducted on territorial land and within the East Bay Migratory Birds Sanctuary under permit NUN-MBS-14-05 granted by Environment and Climate Change Canada.

### Fieldwork

#### Predator-prey indices

Arctic Foxes and jaegers prey upon lemmings, and goose and shorebird nests [11,12]. Glaucous (*Larus hyperboreus*) and Herring Gulls (*Larus smithsonianus*) have been recorded depredating artificial shorebird nests (Young et al. *In Review*; [12]) and regularly depredate goose nests [11,35], but neither have been identified as major predators of real shorebird eggs at our sites, based on time lapse camera footage. Nevertheless, we considered both species as potential predators because they can influence artificial shorebird nest predation [Young et al. *In Review*) and might act as predators of shorebird chicks. Collared Lemmings (*Dicrostonix torquatus*) on Southampton Island experience irregular cycles, but are absent from Coats Island [34].

We generated indices of abundance and activity for predator and prey, respectively, at East Bay Mainland for foxes, jaegers, and lemmings from 2000–2016, and light geese and gulls from 2004–2016 by calculating the number of individuals seen per eight-hour person-day in the field. Individual observers made an effort to avoid repeat counting of predators and prey, but this repeat counting is difficult to avoid across observers. Observational indices are known to be effective at approximating vertebrate abundance [36,37], and are excellent indices in our study because they likely represent a combination of abundances and activity rates, while taking into account non-reproductive individuals. Such a combination is ideal in our study because the impacts of predators on prey likely increase with both the abundance and the activity of predators and prey.

From 2015–2016, we also generated camera predator indices using Plotwatcher Pro^TM^ time-lapse cameras mounted on camouflaged stands placed at the south-eastern corner of each of the study plots (Within, Near, Far, Coats Island). Each camera was positioned facing northwest so as to capture the greatest field of view of the plot (and artificial nests) and then programmed to take a picture every five seconds. This timing likely biases indices of foxes, which spend more time intensively searching for nests in plots, high compared to indices of gulls and jaegers, which move quickly in and out of frame of view, but remains constant among study plots and years. At the end of the field season we reviewed all photographs manually and recorded the species, time, and date that individuals entered and left the field of view of the camera. From 16-June to 20-July of each year we installed permanent cameras within plots (Within: 2 cameras, Near: 2, Far: 3, Coats: 3) situated at increasing distances from the goose colony. Similar to observational predator-prey indices, we generated camera predator indices by calculating the number of predator species seen on camera per eight-hour camera-day. We did not observe lemmings on cameras and, because of the relatively short distance among study sites on Southampton Island, assume that lemming abundance was consistent among them.

#### Shorebird species and nest monitoring

At the study plots on Southampton Island, we monitored the nests of eight tundra-nesting shorebird species: American Golden-Plover (*Pluvialis dominica*), Black-bellied Plover (*Pluvialis squatarola*), Dunlin (*Calidris alpina*), Red Phalarope (*Phalaropus fulicarius*), Ruddy Turnstone (*Arenaria interpres*), Semipalmated Plover (*Charadrius semipalmatus*), Semipalmated Sandpiper (*Calidris pusilla*), and White-rumped Sandpiper (*Calidris fuscicollis*). For each species we used behavioural observations and flushed incubating birds from nests while walking to find nests. We used a Global Positioning System (GPS) to mark the location of each nest (±3m) and placed a wooden tongue depressor ~10m from the nest cup to facilitate subsequent monitoring for nest fate.

Upon discovery of nests, we placed two eggs in warm water and used their degree of flotation to estimate their age and approximate hatch date (±4 days) following [38]. We then revisited each nest weekly throughout the incubation period to document any predation events. Three days prior to expected hatch we visited nests every day to ensure that the fate was documented. We considered nests to be depredated if eggs disappeared before the expected hatch date. For the purposes of this analysis relating to predation, we excluded abandoned nests or any for which we were unsure of their fate.

#### Artificial nest experiment

Artificial shorebird nests comprised four Japanese Quail (*Coturnix japonica*) eggs supplied by CRO Quail Farms Inc., which were placed in a divot in the ground. Quail eggs mimic the size and colouration of *Calidris* shorebird eggs. Survival of artificial and real shorebird nests may differ because of the ability of shorebirds to conceal nests or use distraction displays [39,40], but nevertheless, they have proven to be an effective measure of relative predation risk in several Arctic predation risk experiments [14,39,41]. Our intent with the experiment was therefore to identify *relative* temporal and spatial patterns of risk associated with fluctuations in predator-prey abundance and activity indices rather than absolute levels of predation.

For the experiment, we placed artificial nests in a stratified-random design, with samples evenly distributed among the six dominant habitat types (Dry Heath, Scrub Willow, Sedge Meadow, Moss Carpet, Intertidal, Gravel Ridge; see [14] for descriptions) within the study plots (Within, Near, Far, Coats Island). We marked the location of each artificial nest with a handheld GPS unit and a tongue depressor stuck in the ground within 10m of the nest.

Each year, we deployed and monitored artificial nests during two trials each lasting nine days. We considered artificial nests depredated if at least one egg was taken and successful if the clutch remained intact over the nine-day experiment. Artificial nest experiments were conducted from 28 June– 19 July in 2015, and 28 June– 17 July in 2016. This timing corresponds with the mid- to late-incubation periods for shorebirds breeding at these sites.

### Statistical analyses

#### Temporal trends in predator-prey indices and risk of predation

To identify temporal trends in predator-prey abundance and activity indices at East Bay Mainland we regressed each predator-prey index against time. We also used a general linear model to test for effects of yearly goose indices on gull, jaeger, and fox indices using linear and quadratic fits as well as lemming presence (high or low relative to long-term trends) and an interaction between both.

We related observational predator-prey abundance and activity indices to risk of predation of real shorebird nests over the 13 years (2004–2016) of monitoring at East Bay Mainland that we collected all predator-prey indices, using generalized mixed effects models with a binomial distribution and a logit-link function. Due to the interrelated nature of predator-prey systems, we hypothesised *a priori* that the relationships between predictors would be complex. Consequently, we modeled unanticipated interactions through a best-subsets model building approach using the R package MuMIn [42]. We considered yearly lemming abundance (Lemm) as high or low, compared to the average (0.25) of long-term trends because of the relatively large variation at the site. Observational abundance and activity indices for arctic fox (Fox), jaeger (Jaeger), and gull (Gull) were included as continuous covariates, and the response variable of shorebird nest predation varied between 0% (survived) and 100% (depredated). We tested models that incorporated combinations of individual and additive effects of each predator/prey species as well as interactions between each predator species and lemming abundance. To account for any unexplained non-predator-prey related indices that may co-vary with time we also tested a model with a single variable of Year. We included shorebird species as a random effect in each model, recognizing the consistent differences in nest survival among species e.g., [31,43]. Following [44] we ranked models using Akaike’s information criterion (AIC) and considered models within two AICc of the top model as informative. We also examined the confidence intervals of predictor variables in the top model to determine whether they included zero.

#### Spatial experiment using camera predator-prey indices

We used a general linear model to determine the effect of distance from the goose colony on camera predator indices (response) with plot distance (Within, Near, Far, Coats), predator species (fox, gull, or jaeger), and year (2015 and 2016), and interactions between the three variables as predictors. For the results from the artificial nest experiment we used a generalized linear mixed effects model with a binomial distribution to identify the influence of plot distance from goose colony, year (2015 and 2016), and an interaction between the two on the probability of predation of an artificial nest during the nine-day experiment. In this model we also included trial as a random effect. All statistical analyses were performed in R Version 3.3.1 (R Core Team 2017).

## Results

### Temporal experiment using observational predator-prey indices

**Fig 2:**
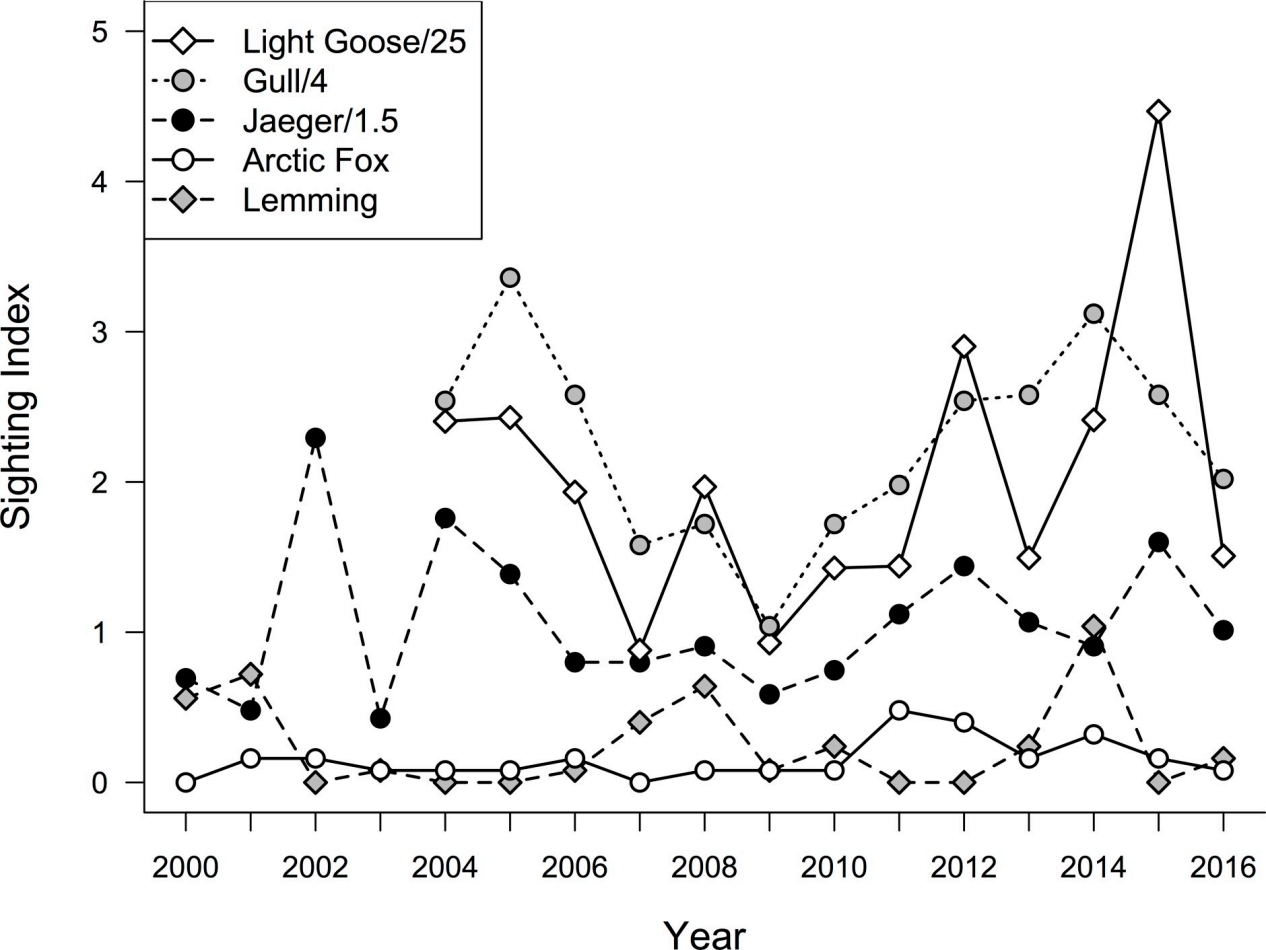
Long-term trends in predator-prey indices at East Bay Mainland. Trends in light goose, gull, jaeger, arctic fox, and lemming sightings per 8-hour person day at East Bay Mainland. Lemming numbers peaked in 2001, 2008 and 2014. In most other years, lemmings were rarely recorded. Sighting indices have been scaled for the purposes of visualization.

**Fig 3:**
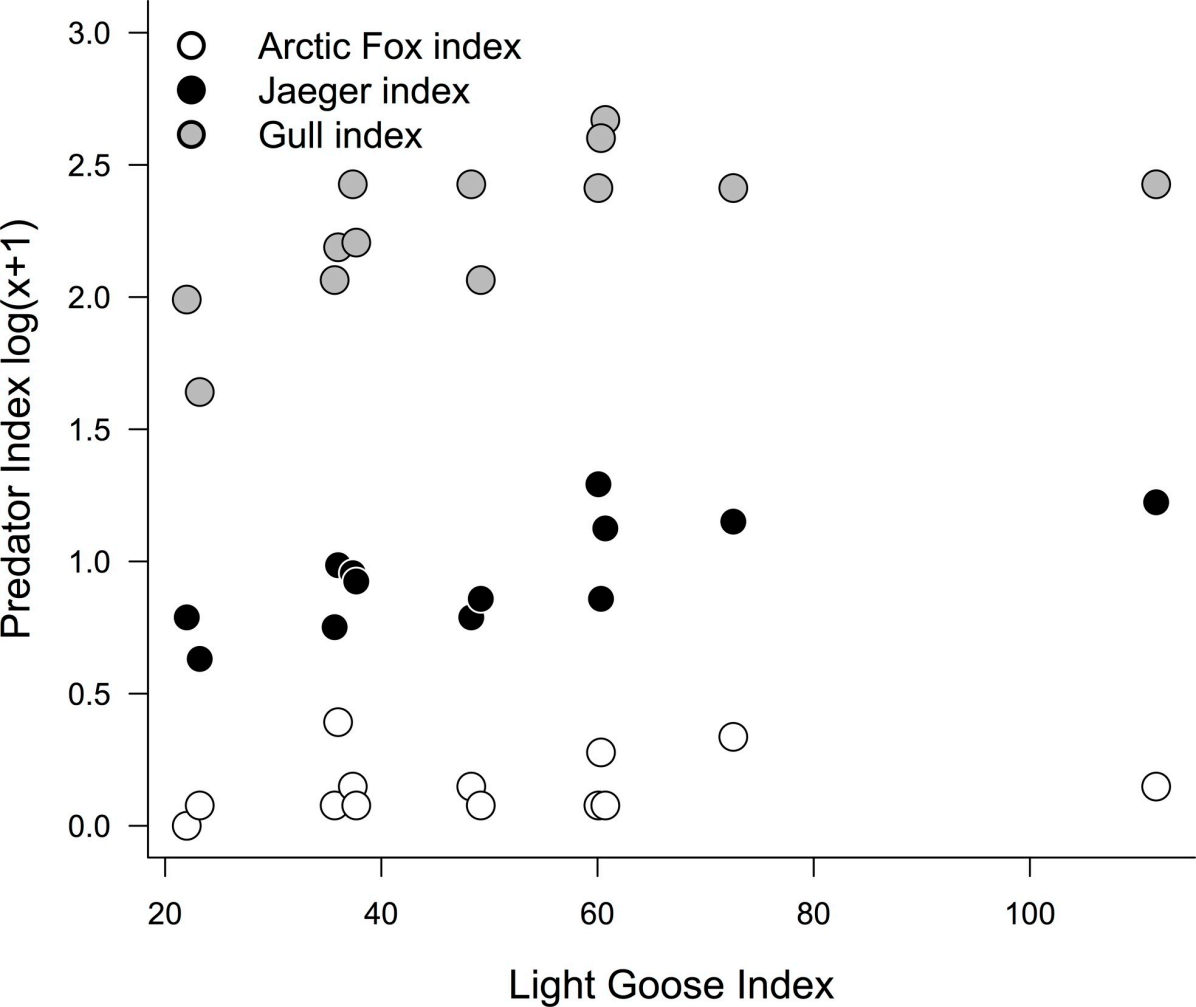
Inter-annual effect of light geese on predator indices. Relationship between yearly observational index (sightings per 8-hour observer day) of light geese, and gulls, jaegers, and arctic foxes for 13 years at East Bay Mainland.

Over the 17-year period at East Bay Mainland we logged 13,453 (mean ± s.d.: 962 ± 434 per year) human observer hours and during 13 years monitored 839 (mean ± s.d.: 69.9 ± 19.1 per year) shorebird nests. During this time period we found evidence of an irregular lemming cycle with peaks occurring in 2001, 2008, and 2014 (mean number of years between peaks = 6.5; Fig 2) and often no lemmings observed during low years. Over the 13–16 years where all predator-prey observations were recorded individual predator-prey indices fluctuated among years but no individual models described significant linear increases or decreases (all p’s > 0.05; Fig 2). We found a positive quadratic effect of goose abundance on gull and jaeger indices, but little effect on foxes (F_2, 30_: 6.86, p < 0.01; Fig 3). Lemming presence (high or low) had no effect on any predator indices (p > 0.05).

**Fig 4:**
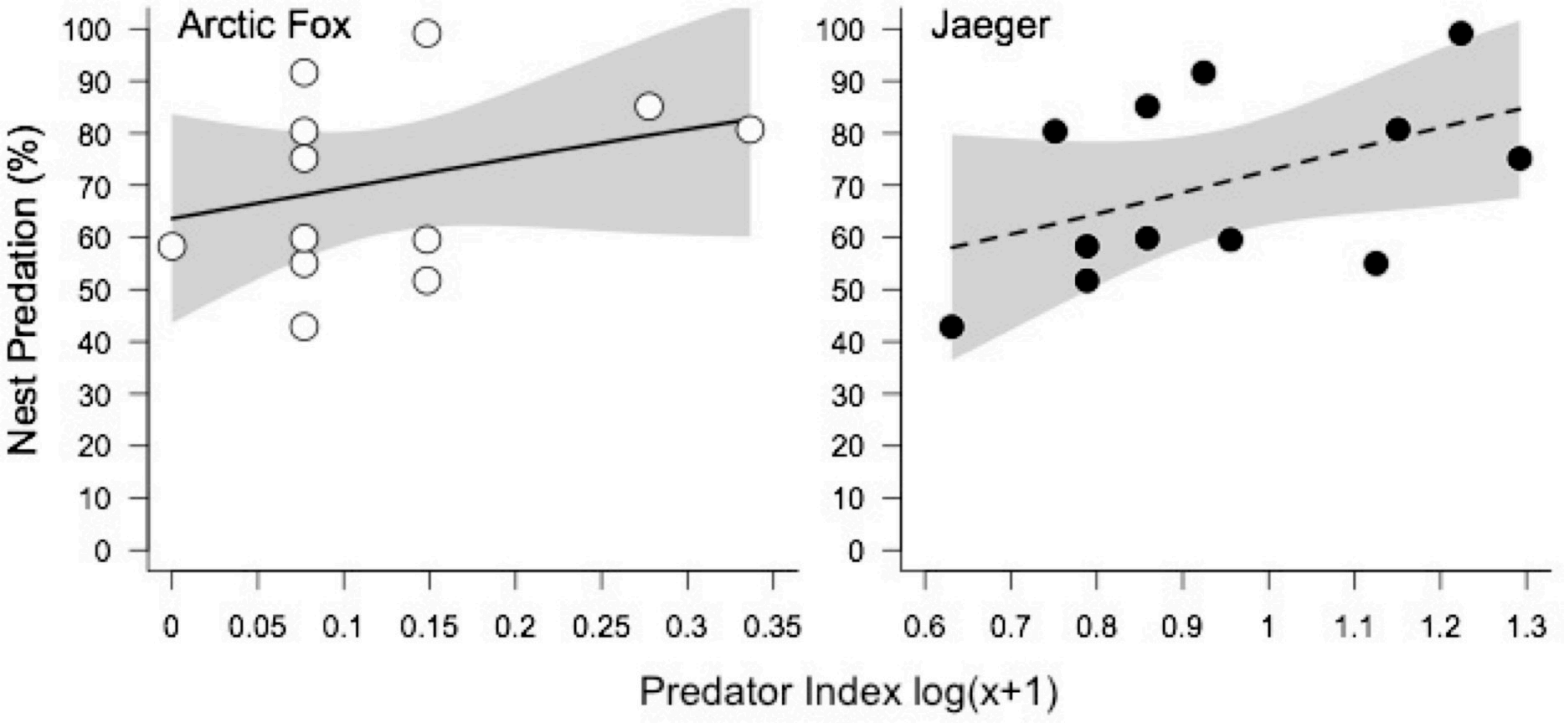
Shorebird nest survival probability. Effects of Arctic fox and Jaeger indices on the probability of predation of shorebird nests over 13 years.

Over the 13 years, shorebird nest predation at East Bay Mainland was highest in 2016 (96%) and lowest in 2007 (49%). The top models for predicting nest predation included lemming, fox, jaeger, and gull indices, but no interactions between any variables (Table 1). Fox abundance and activity indices were informative in the top models and positively influenced the predation rate of shorebird nests (relative importance: 0.96; Table 2; Fig 4) while jaeger indices were also relatively important (0.72).

**Table 1:** Model selection.

Model	K	AIC_c_	ΔAIC_c_	w_i_
Lemm + Fox + Jaeger	5	88.59	0.00	0.65
Lemm + Fox + Jaeger + Gull	6	89.85	0.27	0.35
Null	2	98.46	9.87	0.00

Best subsets model selection (AIC < 2, plus null) results for the influence of predator-prey indices on probability of predation of real shorebird nests.

**Table 2:** Parameter estimates for top models.

Variable	$\hat{B}$	SE	*P*	95% CI
Lemm	-0.99	0.65	0.13	-2.29, 0.30
Fox	13.38	5.93	0.03	1.59, 25.17
Jaeger	1.46	0.78	0.06	-0.08, 3.00
Gull	-0.19	0.18	0.32	-0.55, 0.18

Parameter estimates for top logistic models best describing probability of predation of real shorebird nests.

### Spatial experiment using camera predator-prey indices

**Fig 5:**
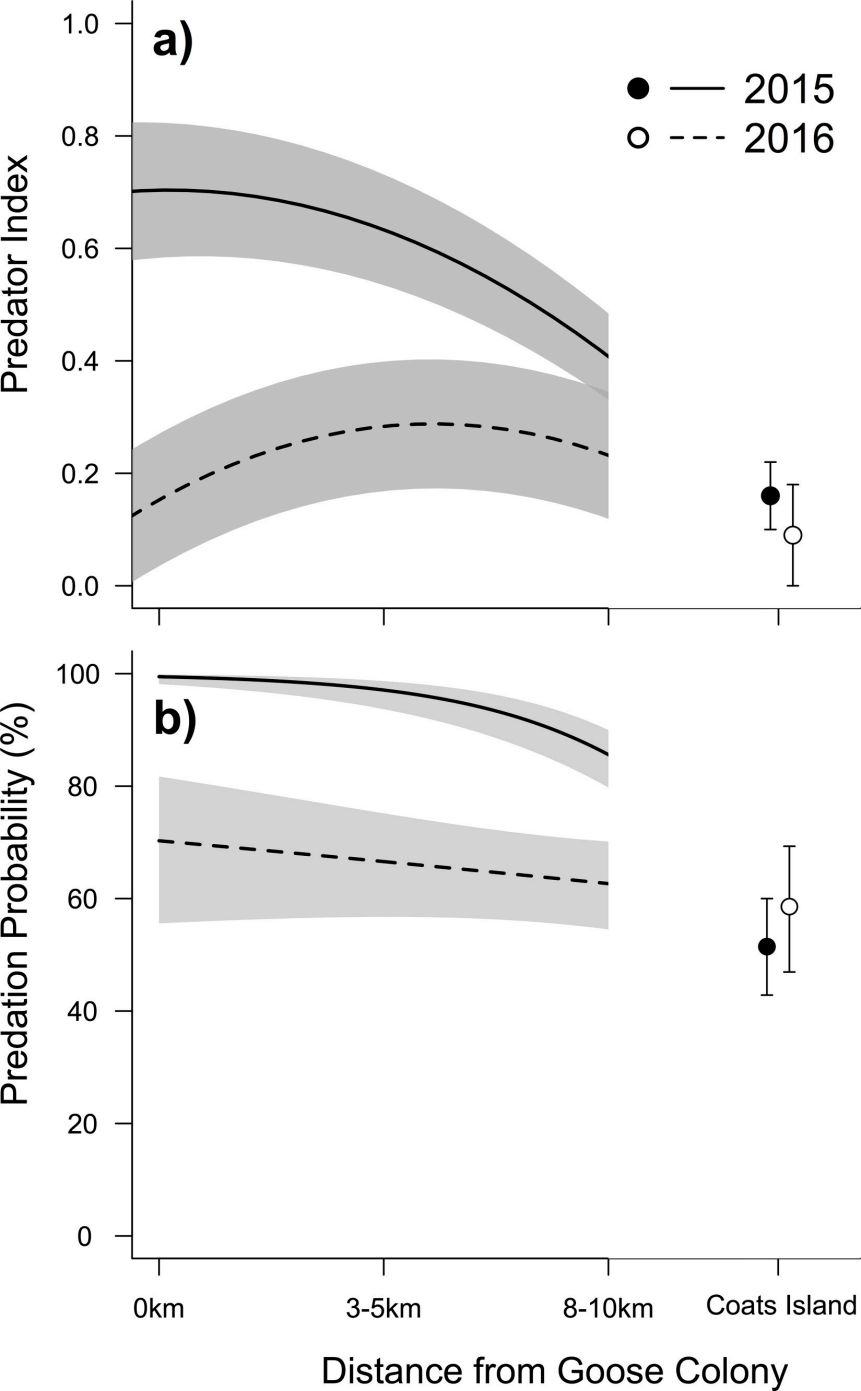
Relationship between distance from a goose colony and a) total of all predator indices, and b) probability that artificial nests were depredated during the nine-day experiment in 2015 and 2016.

During the two years of camera monitoring within the four study plots situated at increasing distances from the goose colony, we recorded 413 independent predator sightings during 9,613 hours of camera monitoring. We also deployed 530 artificial nests. Overall camera predator indices varied significantly (F_2, 12_: 20.41, p < 0.001) with gull indices being the highest (0.23 sightings per camera day), followed by fox (0.06) and jaeger indices (0.02). Camera predator indices were negatively related to distance from the goose colony (F_1, 12_: 13.31, p < 0.001), but this effect varied by year (F_1, 12_: 5.24, p < 0.05; Fig 5A). The predation probability of artificial nests varied by year (F_1, 522_: 15.68, p < 0.001; Fig 5B) with it lower in 2016 compared to 2015 and was inversely related to distance from the goose colony (F_1, 522_: 77.22, p < 0.001), during one year (F_1, 522_: 14.26, p < 0.001).

## Discussion

In Arctic and sub-Arctic systems where food availability is seasonal and unpredictable, generalist predators may exhibit functional and numerical responses, thereby affecting the survival of multiple prey species. Recent rises in populations of light geese and alterations in cycles of lemmings may therefore influence the cyclic dynamics of generalist predators and indirectly influence the risk of predation and probability of survival of incidental prey such as shorebirds. Over 13 years we found that gull and jaeger abundance and activity indices were positively associated with yearly goose indices, regardless of lemming abundance and that during one of two years of camera monitoring all predator indices were negatively related to distance from goose colony. Long-term monitoring of shorebird nests indicated that predation probability was positively related to fox and jaeger indices. In addition, artificial shorebird nest predation probability, during the two year experiment, was negatively associated with distance from goose colony. These results add to our understanding of variations in predator-prey relationships among Arctic study sites and suggest that, in some areas, light goose presence *has the potential to* indirectly affect the risk of predation for shorebirds by influencing the temporal and spatial distribution, and activity of their shared predators.

### Predator indices

Functional and numerical responses of generalist Arctic predators to their primary prey, lemmings, have been well-documented [11,13,45]. The strength of these relationships may vary by site [46] and depend on the presence of allochthonous resources such as geese [10,23,47]. Over a 17-year period, we found no systematic changes in indices of predators or prey at our East Bay Mainland site, 10 km away from a goose colony. Yearly indices of geese did however positively influence indices of two potential nest predators, gulls and jaegers, while lemming abundance had no measurable effect on the abundance of any predators in our time series. When food is abundant, jaegers may have more nests, produce larger clutches, and experience elevated breeding success [2,45]. Abundant goose eggs and goslings can also contribute to higher growth of gull chicks [48], potentially bolstering some populations [49]. It is therefore possible that at East Bay Mainland, during years when goose eggs and goslings are abundant in the nearby colony, gulls and jaegers are experiencing higher overall reproductive success and/or exhibiting elevated activity rates in response to the demands of their offspring.

We also found all predator indices were higher within and near the goose colony compared to far from the colony, but this relationship varied between the two years and may be the result of varying goose abundance and colony size. In 2015 goose, gull, jaeger, and fox indices at East Bay Mainland were 2.96, 1.23, 1.58, and 2.00 times higher, respectively, than in 2016. This trend is consistent with the long-term positive association between geese and predator indices. Inter-annual fluctuations in the size of goose breeding populations are not uncommon [26,50] and may reflect variations in breeding effort due variation in temperature, snow, and other weather conditions on the breeding grounds [51]. The boundaries of breeding colonies can also vary inter-annually [25,52]. These factors could have influenced the number and distribution of geese in 2015 vs. 2016, potentially influencing distribution of nest predators captured in our camera indices.

Regardless of this inter-annual variation, generalist predator indices at the three sites on Southampton Island in both years were substantially higher than on Coats Island where both breeding light geese and lemmings are absent. Lamarre et al., [52] documented aggregative responses of arctic fox and aerial predators to a goose colony on Bylot Island, Nunavut; this effect was pronounced for foxes when lemmings were scarce. Parasitic jaeger activity can also be elevated within light goose colonies during non-lemming peak years [11,35]. Although we found no effect of lemming abundance on predator indices in our long-term data at East Bay Mainland, over more years of monitoring, particularly within the goose colony, an influence of lemmings on predator indices may still emerge, and show that lemming peaks could be contributing to some of the variation in our predator indices.

Goose eggs and goslings can sustain elevated predator populations [48] in the absence of abundant and regular lemming cycles and may therefore be responsible for the higher indices on Southampton Island than on Coats Island where predators are limited to depredating patchily distributed waterfowl and shorebird nests. Overall, these results highlight the numerical and aggregative responses of generalist predators to an increasing goose colony and the importance of goose colonies in buffering predators against lemming crashes [53].

### Risk of predation

Functional and numerical responses of predators to preferred prey can influence the risk of predation for incidental prey [13,54,55]. Across 13 years of nest monitoring on Southampton Island, predation rates of real shorebird nests was positively correlated with our fox and jaeger indices, but lemmings had no effect. Individually, foxes and jaegers increased nest predation probability by ~5% per 0.1 index increase. During years when both predator indices are elevated, predation rates of shorebird nests could reach 91%, an increase of ~42% from low predator years. Considering the average number of nests found per year at our sites is 70, this equates to only 6 successful shorebird nests, excluding abandonment.

In a large-scale multi-year analysis across 14 shorebird species and 17 sites, Weiser et al. [43] also reported no effect of arvicoline rodents on survival of shorebird nests. Furthermore, this study found evidence of a non-linear fox-related decline in Western Sandpiper (*Calidris mauri*) daily nest survival probability of 0.08 between maximum and minimum fox abundances, further supporting our results and highlighting the variation in predator-prey relationships among sites.

Over a shorter temporal period (two years) we also found a negative relationship between distance from goose colony and predation rate of artificial shorebird nests that corresponded to the spatial patterns in camera predator indices. This effect was most pronounced in 2015 when overall predation rate was higher than in 2016.

In a five-year study on Bylot Island, in the Canadian high arctic, [52] found a similar relationship between distance from goose colony and the probability of predation rate of artificial nests, however, this effect was modulated by lemming abundance. At the same site, [17] reported survival of artificial shorebird nests was positively related to lemming abundance, but may be reduced in the areas with higher densities of goose nests that foxes are attracted to [13]. Although our artificial nest experiment was confined to two years, one with moderate numbers of lemmings and one without, our long-term data at one site indicated no effect of lemmings on probability of predation of real shorebird nests. It is possible that distance from goose colony is interacting with lemming abundance at a smaller scale that we may not be detecting in our long-term data at the site ~10km from the colony. However, [52] found effects on artificial nest predation greater than ~10km away suggesting our scale may be adequate. Overall, these studies support our results and further highlight that predator-prey relationships may vary among sites and regions as indicated by the lower predation rate [elevated survival probability) in the presence of nesting geese reported elsewhere [19,20].

### Population-level consequences and sub-lethal effects

The spatio-temporal goose-related trends in predator indices we report have the potential to drive nesting decisions, densities, and reproductive success of shorebirds that breed in northern latitudes in response to their perceived predation risk [39]. When faced with locally elevated predator abundances, birds may choose to nest and suffer elevated predation [15], abandon, or forego nesting [56]. Goose-augmented predator communities have already been implicated in the locally depressed densities of shorebirds around goose colonies [52,57,58]). Goose-induced habitat alteration is also well-documented [50,57,59] and may decrease the availability of vegetative concealment that influences nest survival for some species [14,15,60], potentially compounding the effects of geese. Furthermore, non-consumptive effects of predators such as elevated stress, fewer or smaller eggs, and increased nest recesses in response to elevated perceived risk of predation have been reported in other species [61–63] and warrant investigation in the context of Arctic-nesting shorebirds.

### Conclusions

The opposing population trends of light geese and shorebirds, and significant overlap in range and habitat use [64] suggest the potential for geese to be influencing the reproductive success of sympatric shorebirds. We found evidence of complex interactions between geese and shorebirds mediated by generalist predators. Light geese bolstered jaeger and to a much lesser extent arctic fox populations, which negatively affected shorebird nest survival. Predator activity was elevated around the goose colony, but exhibited inter-annual variation, which translated to variation in survival of artificial nests. The resulting elevated risk of predation around the colony could be influencing shorebird nesting strategies and ultimately reproductive success. The potential for population-level consequences is further compounded by goose-induced habitat alteration that can reduce vegetative concealment [57] perhaps increasing predation risk for some shorebird species. Our study highlights the importance of identifying interactions between geese and shorebirds, which may vary by site and prey abundance, and how the effect of light goose colonies on shorebirds can extend further than any habitat-related effects close to the colony.
